# A 192 bp ERV fragment insertion in the first intron of porcine TLR6 may act as an enhancer associated with the increased expressions of *TLR6* and *TLR1*

**DOI:** 10.1186/s13100-021-00248-w

**Published:** 2021-08-18

**Authors:** XiaoYan Wang, Zixuan Chen, Eduard Murani, Enrico D’Alessandro, Yalong An, Cai Chen, Kui Li, Grazia Galeano, Klaus Wimmers, Chengyi Song

**Affiliations:** 1 College of Animal Science & Technology, Yangzhou University, Yangzhou, 225009 Jiangsu China; 2 Leibniz Institute for Farm Animal Biology (FBN), 18196 Dummerstorf, Germany; 3 Department of Veterinary Science, Unit of Animal Production, University of Messina, 98168 Messina, Italy; 4 Institute of Animal Science, Chinese Academy of Agricultural Sciences, 100081 Beijing, China

**Keywords:** Pig, Retrotransposon, RIP, ERV, *TLRs*, *TLR6*, Enhancer, Expression, Polymorphism

## Abstract

**Background:**

Toll-like receptors (TLRs) play important roles in building innate immune and inducing adaptive immune responses. Associations of the *TLR* genes polymorphisms with disease susceptibility, which are the basis of molecular breeding for disease resistant animals, have been reported extensively. Retrotransposon insertion polymorphisms (RIPs), as a new type of molecular markers developed recently, have great potential in population genetics and quantitative trait locus mapping. In this study, bioinformatic prediction combined with PCR-based amplification was employed to screen for RIPs in porcine *TLR* genes. Their population distribution was examined, and for one RIP the impact on gene activity and phenotype was further evaluated.

**Results:**

Five RIPs, located at the 3' flank of *TLR3*, 5' flank of *TLR5*, intron 1 of *TLR6*, intron 1 of *TLR7*, and 3' flank of *TLR8* respectively, were identified. These RIPs were detected in different breeds with an uneven distribution among them. By using the dual luciferase activity assay a 192 bp endogenous retrovirus (ERV) in the intron 1 of *TLR6* was shown to act as an enhancer increasing the activities of *TLR6* putative promoter and two mini-promoters. Furthermore, real-time quantitative polymerase chain reaction (qPCR) analysis revealed significant association (*p* < 0.05) of the ERV insertion with increased mRNA expression of *TLR6*, the neighboring gene *TLR1*, and genes downstream in the TLR signaling pathway such as *MyD88 (*Myeloid differentiation factor 88*)*, *Rac1* (Rac family small GTPase 1)*, TIRAP* (TIR domain containing adaptor protein)*, Tollip* (Toll interacting protein) as well as the inflammatory factors *IL6* (Interleukin 6)*, IL8* (Interleukin 8), and *TNFα* (Tumor necrosis factor alpha) in tissues of 30 day-old piglet. In addition, serum IL6 and TNFα concentrations were also significantly upregulated by the ERV insertion (*p* < 0.05).

**Conclusions:**

A total of five RIPs were identified in five different *TLR* loci. The 192 bp ERV insertion in the first intron of *TLR6* was associated with higher expression of *TLR6*, *TLR1*, and several genes downstream in the signaling cascade. Thus, the ERV insertion may act as an enhancer affecting regulation of the TLR signaling pathways, and can be potentially applied in breeding of disease resistant animals.

**Supplementary Information:**

The online version contains supplementary material available at 10.1186/s13100-021-00248-w.

## Background

Toll-like receptors (TLRs) play vital roles in innate and adaptive immune responses due to their ability to recognize different types of pathogens and associated molecular patterns and activate immune-related signaling pathways [1]. Polymorphisms in *TLR* loci and their influence on either susceptibility or resistance to major human infectious diseases, including tuberculosis, leishmaniasis, malaria, filariasis and some autoimmune endocrine diseases, have been reported extensively [2–4]. It has been reported that C1174T substitution in *TLR5* resulting in a stop codon polymorphism causes significantly lower levels of proinflammatory cytokines in comparison to individuals with the wild-type genotype and that the *TLR5* stop codon polymorphism is associated with protection from the development of systemic lupus erythematosus [5]. The missense mutation rs5743618 in *TLR1*, specific for Europeans, can change the expression of 81 genes involved in the inflammatory response [6]. Chikungunya patients with rs179010-CC genotype of *TLR7* showed significantly higher interferon alpha 1 (IFNα) level, which might act as potential prognostic biomarkers for predicting Chikungunya susceptibility [7]. It is commonly accepted that *TLRs* are important candidate genes for some human immune diseases.

For domesticated farm animals, *TLRs* also have been suggested as the most promising candidate genes for improvement of immune response or disease resistance by molecular breeding [8]. Genetic variants of *TLRs* associated with cattle mastitis, mycobacterial infection, and paratuberculosis have been identified [9–11]. In pigs, ten *TLRs* were annotated in the genome, and a number of studies reported on SNP screening of the porcine *TLRs* and their expression patterns in immune response-related organs [12]. C506W substitution in *TLR4* cDNA in Japanese segregating in wild boar populations caused loss of ability to induce nuclear factor kappa B subunit (NF-κB) activation after lipid A stimulation [13]. Association between SNPs in *TLR4* and *TLR5* with transcript abundance of cytokine genes indicates that these SNPs are related to the modulation of the cytokine mediated immune response [14, 15]. Recently, genetic variation in all 10 *TLRs* across 11 pig breeds was screened by using targeted sub-genome enrichment and high-throughput sequencing, and 306 SNPs were discovered [16]. In another report, a total of 136 SNPs was obtained by sequencing *TLR1*, *TLR2*, *TLR6*, *TLR3*, *TLR7*, and *TLR8* genes, and a variant G376A( Ala126Thr) in *TLR2* was identified to be under positive selection in pigs of European origin. A 3D crystal structure analysis revealed that this SNP (G376A) may affect ligand binding, indicating that TLR2 may contribute to responses to bacterial pathogens, and play an important role in adaptation of pigs to pathogens [17]. It has been suggested that piglets with the T allele of a C1205T substitution in *TLR5* cDNA exhibit impaired resistance to *Salmonella typhimurium* infection [18]. So far, all association studies of *TLRs* with diseases susceptibility were based on SNPs, however reports on retrotransposon insertion polymorphisms in *TLRs* and their genetic effects are not available.

Retrotransposons are important components of plant and animal genomes, accounting for nearly half of the mammalian genomes [19, 20], and can mobilize themselves to new genomic locations and generate polymorphic insertions. Retrotransposons can be classified into LTR (Long Terminal Repeat elements, including endogenous retrovirus, ERV) and non-LTR families (including LINE, Long Interspersed Nuclear Elements; and SINE, Short Interspersed Nuclear Elements) [21]. For a long time, transposable elements including retrotransposon have been considered as genomic parasites and ‘junk DNA’ [22, 23]. However, mounting evidence suggests that retrotransposons contribute to genome architecture and evolution, and even maintenance of three-dimensional chromatin organization in mammals [20, 24–26].

Retrotransposon insertion polymorphisms (RIPs) have been applied as molecular markers to study genome evolution and genetic diversity in plants [27, 28]. In humans, RIPs have been identified as causative mutations for some diseases [29]. Genome-wide association studies revealed an intronic Alu insertion polymorphism in *CD58* gene associated with multiple sclerosis risk possibly due to altering its mRNA splicing [30, 31]. In domestic animals, RIPs also have been used for evolution and population genetic analysis in sheep [32], cat [33], chicken [34], and rabbit [35]. Several RIPs have been associated with phenotypic variation in farm animals, such as an ERV insertion in the 5’ flanking region of *SLCO1B3* causing blue eggshell by promoting the expression of the *SLCO1B3* gene in the uterus (shell gland) of the oviduct in chicken [36, 37], the henny feathering allele harboring an insertion of an intact avian ERV at the 5'end of *CYP19A1* [38], and the SINE insertions in the follicle stimulating hormone beta (*FSHβ)* and the protein disulfide isomerase associated 4 (*PDIA4*) genes associated with litter size variations in pigs [39, 40].

In the present study, the contribution of RIPs to the structural variations of *TLR* genes, the breed distribution of these RIPs and the genetic effects of one RIP were investigated. We identified five RIPs, each in a different *TLR* gene, and our data suggest that one RIP may play a role in the regulation of the TLR signaling pathway by acting as an enhancer. These findings will contribute to the understanding of the role of RIPs in shaping the pig genomic and genetic variation, and one RIP may be useful for molecular breeding to improve disease resistance in the pig.

## Results

### Five RIPs generated by retrotransposon insertions in the pig *TLR* gene cluster

**Fig. 1:**
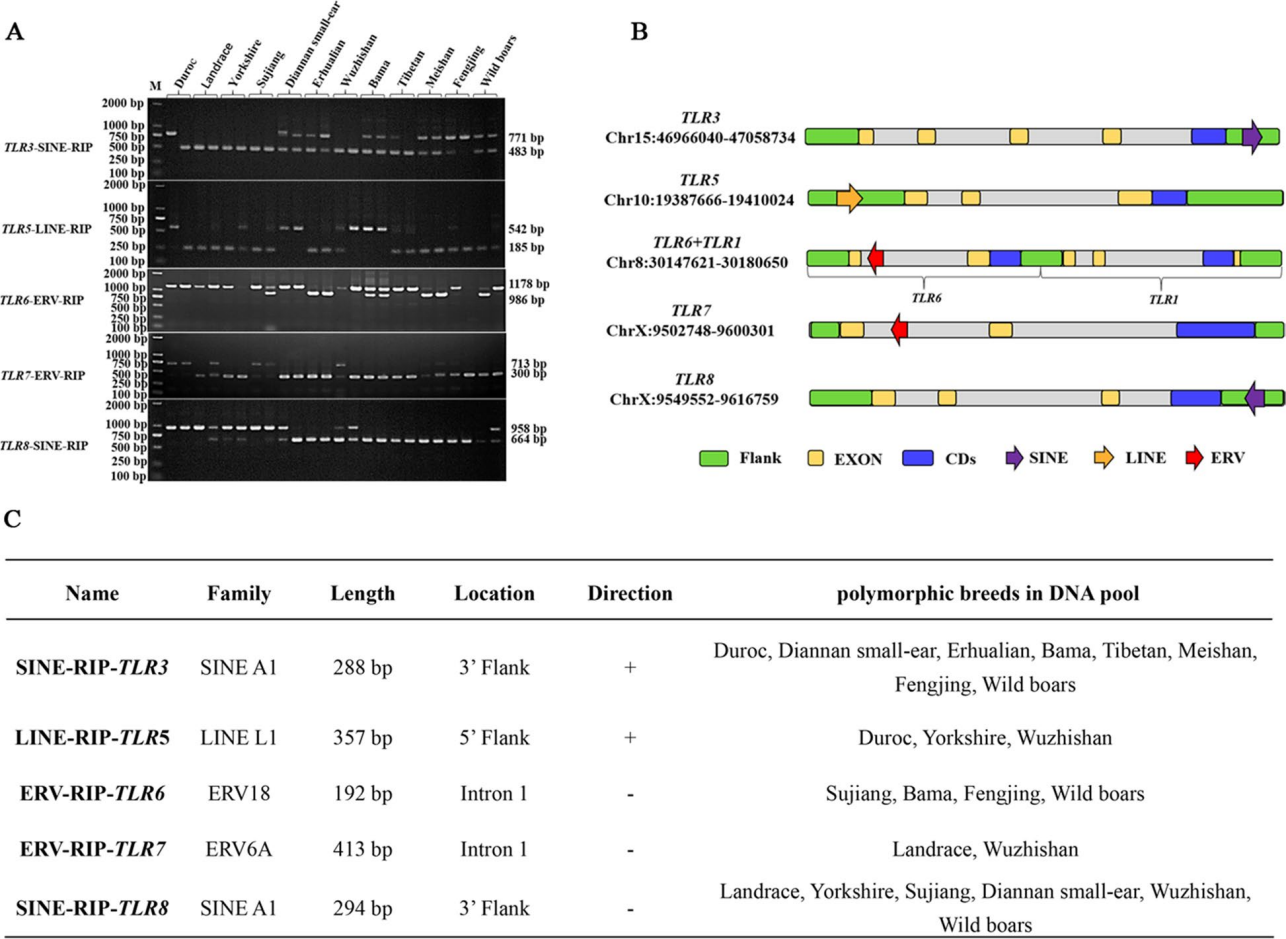
PCR identification and characteristic of RIP in *TLRs. ***a** RIPs were identified in DNA pool by PCR; **b** Location of RIPs in *TLRs*; **c** Characteristic of RIPs in *TLRs*

Ten *TLR* genes and their flanking sequences from sixteen assembled pig genomes, representing lean type pigs (Cross-breed of Yorkshire/Landrace/Duroc, Duroc, Landrace, Yorkshire, Pietrain, Berkshire, and Hampshire), fat type pigs (Rongchang, Meishan, Bamei, and Jinhua), and miniature pigs (Bama, Wuzhishan, Tibetan, Goettingen, and Ellegaard Gottingen) were used to screen for structural variations by sequence alignment using the ClustalX program [41]. In total, we identified 53 large structural variations (SVs, defined as variants more than 50 bp and less than 1000 bp long) or large frameshift variants (more than 1000 bp long), and 32 of them were predicted as RIPs, including 15 SINE, 11 ERV, and 6 LINE RIPs, which were summarized in additional file 1 (Table S1). Then all these predicted RIPs were further experimentally evaluated by PCR screening of pooled DNA samples of 11 domesticated pig breeds and wild boar using specific primer pairs spanning the insertions. Five RIPs, including two SINE RIPs, two ERV RIPs and one LINE RIP, were confirmed by the PCR screening (Fig. 1A). All these RIPs were further confirmed by TA cloning and Sanger sequencing. One 288 bp and one 294 bp SINE RIPs in the 3’ flanking sequence of *TLR3* and *TLR8* were detected, respectively. Moreover, a single 357 bp LINE RIP in the 5’ flanking region of *TLR5* was found as well as one 192 bp and one 413 bp ERV RIPs in first intron of *TLR6* and *TLR7*, respectively. We named those insertions as *TLR*3-SINE-RIP, *TLR5*-LINE-RIP, *TLR6*-ERV-RIP, *TLR7*-ERV-RIP, and *TLR8*-SINE-RIP, respectively (Fig. 1B and 1C).

### RIP distribution in different pig breeds

For the confirmed RIPs (Fig. 1A) their segregation was examined in individual samples of the twelve different breeds. Furthermore, *TLR6*-ERV-RIP was evaluated in three additional populations (Landrace and Yorkshire from Germany and Sicilian Black from Italy). Detailed information (including the number of animals used, breed origins, and genotype and allele frequency) for each breed is summarized in Table 1. The results of PCR genotyping confirmed that all RIPs in these breeds are polymorphic, i.e. both alleles (RIP^±^) were detected, except for *TLR6*-ERV-RIP which proved to be monomorphic in Sicilian Black pigs from Italy, and Landrace and Yorkshire from Germany. For the most part genotype distribution of the RIPs was in Hardy–Weinberg equilibrium (*p* > 0.05), while *TLR3*-SINE-RIP in Meishan, *TLR6*-ERV-RIP in Sujiang, Erhualian and Fengjing, *TLR7*-ERV-RIP in Landrace, *TLR8*-SINE-RIP in Wuzhishan deviated from the Hardy–Weinberg equilibrium (*p* < 0.05). The SINE^+/+^ genotype of *TLR3*-SINE-RIP in Duroc and Tibetan, the ERV^−/−^ genotype of *TLR6*-ERV-RIP in Fengjing, and the ERV^+/+^ genotype of *TLR7*-ERV-RIP in Landrace and Wuzhishan were not detectable. In most breeds the RIPs displayed moderate polymorphic information content (PIC, ranging from 0.239 to 0.375), except for Duroc and Tibetan, where *TLR3*-SINE-RIP shows low PIC values (low than 0.150).

**Table 1:** Genotype and allele frequency of five RIPs in the RIP-polymorphic breeds

RIP	Breed	N	Genotype/%			Allele/%		Hardy–Weinberg/P	PIC
			** + / + **	** ± **	**-/-**	** + **	**-**		
***TLR3-*SINE-RIP**	Duroc	24	0	16.67	83.33	8.33	91.67	0.656	0.141
	Erhualian	24	25.00	45.83	29.17	47.92	52.08	0.689	0.375
	Bama	30	3.33	60.00	36.67	33.33	66.67	0.552	0.346
	Tibetan	35	0	17.14	82.86	8.57	91.43	0.579	0.144
	Meishan	24	37.50	29.17	33.33	52.08	47.92	0.042	0.375
	Fengjing	23	47.83	39.13	13.04	67.39	32.61	0.599	0.343
***TLR5-*LINE-RIP-**	Duroc	24	4.17	62.50	33.33	35.42	64.58	0.073	0.353
	Yorkshire	24	8.33	41.67	50	29.17	70.83	0.967	0.328
	Wuzhishan	24	29.17	50.00	20.83	54.17	45.83	0.973	0.373
***TLR6-*ERV-RIP**	Sujiang	163	52.76	20.86	26.38	63.19	36.81	1.89^e−12^	0.357
	Erhualian	36	27.78	22.22	50.00	38.89	61.11	0.001	0.362
	Bama	43	44.19	39.53	16.28	63.95	36.05	0.350	0.355
	Fengjing	24	41.67	58.33	0	70.83	29.17	0.044	0.328
	Yorkshire (German)	31	100	0	0	100	0	1	0
	Sicilian black (Italy)	30	100	0	0	100	0	1	0
	Landrace (German)	32	100	0	0	100	0	1	0
***TLR7-*ERV-RIP**	Landrace	18	0	83.33	16.67	41.67	58.33	0.002	0.368
	Wuzhishan	23	0	43.48	56.52	21.74	78.26	0.183	0.282
***TLR8-*SINE-RIP**	Landrace	24	75.00	16.67	8.33	83.33	16.67	0.050	0.239
	Yorkshire	24	62.50	29.17	8.33	77.08	22.92	0.393	0.291
	Sujiang	24	50.00	45.83	4.17	72.92	27.08	0.432	0.317
	Wuzhishan	23	43.48	26.09	30.43	56.52	43.48	0.024	0.371

Only the breeds displaying polymorphic RIPs in Fig. 1A were used for further RIP distribution evaluation by increasing individuals except Diannan small-ear pig, Sicilian black from Italy and Landrace and the Yorkshire from Germany were used for *TLR6*-ERV-RIP evaluation. Polymorphic information content (PIC) was measured by using the formula as described in Materials and Methods. The Hardy–Weinberg was detected by and the *p* < 0.05 indicates that the RIP distribution is deviated from the Hardy–Weinberg equilibrium

### Evidence of enhancer activity of the 192 bp ERV insertion

**Fig. 2:**
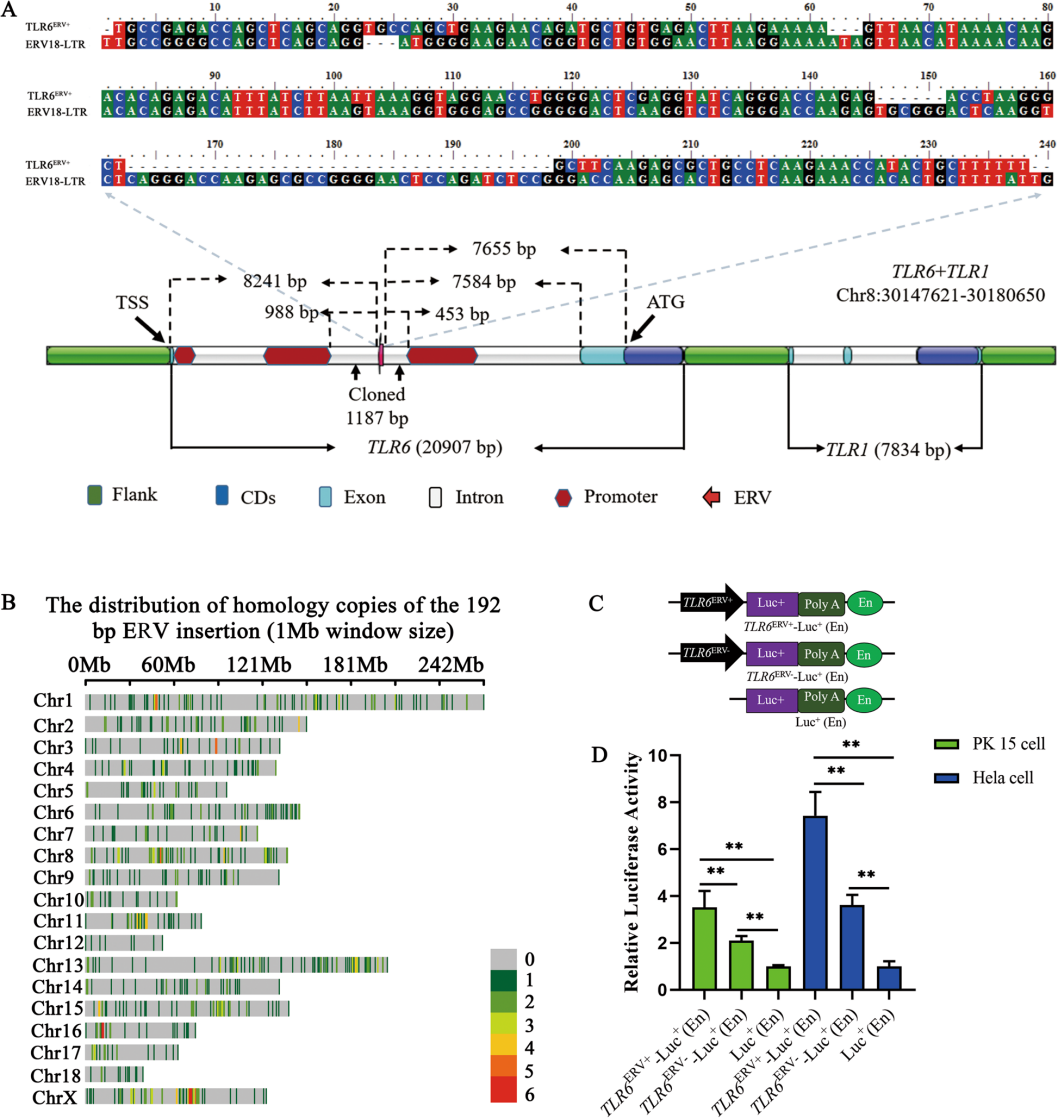
Activity of 192 bp ERV based on the luciferase activity assays. **a** Sequence analysis and promoter prediction of pig *TLR6*; **b** The distribution of homology copies of the 192 bp ERV insertion (1 Mb window size) in genome; **c** A schematic diagram of the recombinant vector using pGL3-basic vector. En: SV40 enhancer; **d** Results on the luciferase activity assays. ** showed *p* < 0.01 between groups

**Fig. 3:**
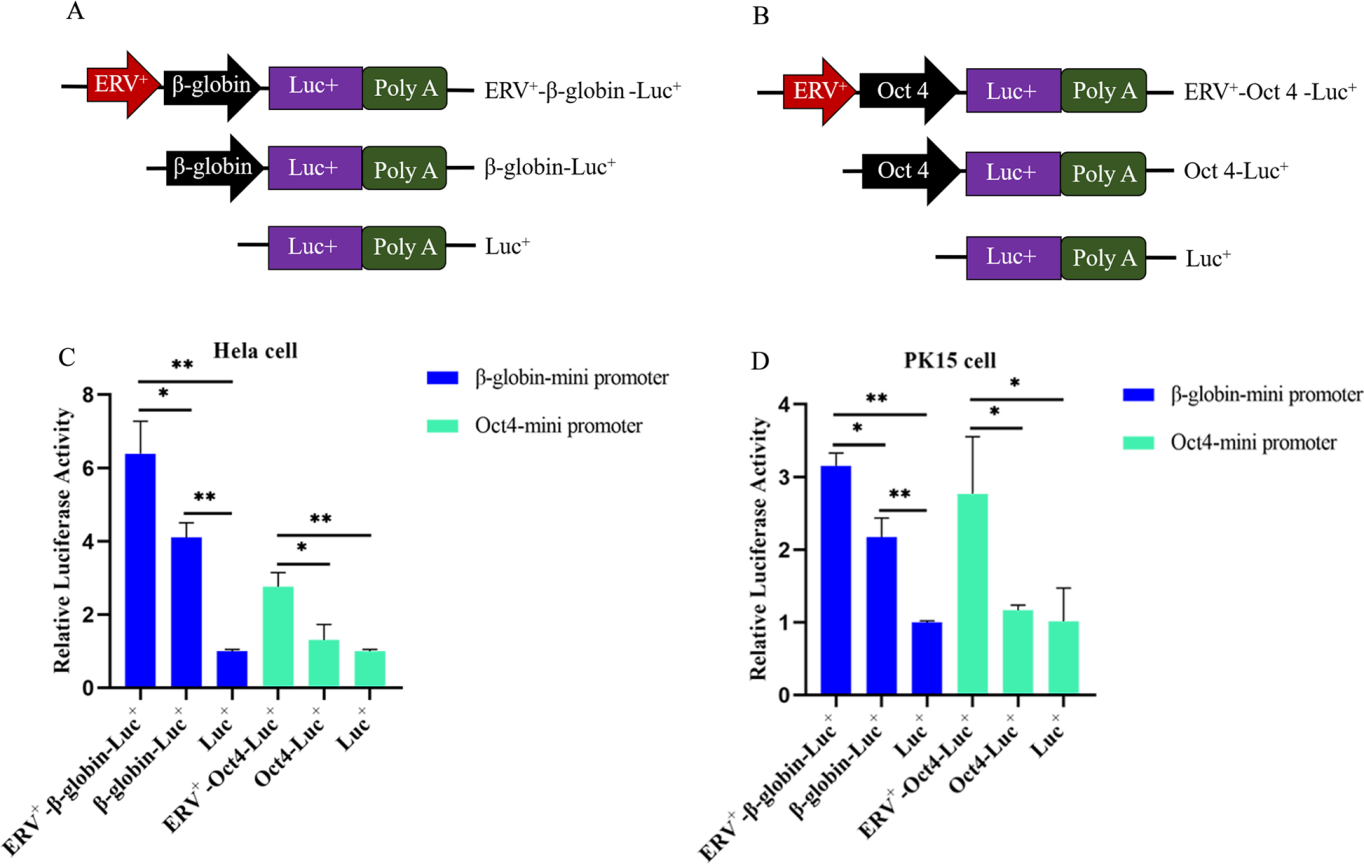
Impact of ERV on the activities of mini-promoters. **a** Plasmid diagram of the recombinant vector with mini-romoter β-globin using pGL3-basic vector; **b** Plasmid diagram of the recombinant vector with mini-promoter Oct4 using pGL3-basic vector; **c** Impact of ERV insertion on the activities of β-globin and Oct4 mini-promoter in PK15; **d** Impact of ERV insertion on the activities of β-globin and Oct4 mini-promoters in Hela cell. * showed *p* < 0.05; ** showed *p* < 0.01

Both of *TLR6* and *TLR7* genes contain an ERV insertion in the first intron, and further analysis revealed that the 192 bp ERV insertion in intron 1 of *TLR6* resides between two putative promoters (988 bp upstream and 453 bp downstream to the ERV insertion), with high prediction scores (> 1) by Promoter 2.0, BDGP, and ENCODE. The 192 bp ERV was a truncated LTR fragment of ERV18 (Fig. 2A), which was identified as a beta ERV, and is located close to the human HERV-K as revealed by phylogenetic analysis [42]; only one intact copy (100% identity and 100% coverage), but about 800 homologous copies (sequence identity > 85% and coverage > 70%) of the 192 bp ERV insertion were identified in the porcine genome by BLAST search (Fig. 2B). The sequence between nucleotides 20–50 and 90–120 of the 192 bp ERV fragment is enriched for transcription factor binding motifs. To further evaluate the potential involvement of the ERV insertion in the regulation of promoter activity of *TLR6*, alternative genomic fragments, either containing (1187 bp) or lacking (995 bp) the 192 bp ERV insertion, were cloned into a luciferase reporter vector (pGL3-basic) respectively (Fig. 2C), and then submitted to luciferase activity evaluation. The dual luciferase activity assay revealed that the genomic fragment lacking the ERV insertion allele (995 bp) displayed considerable promoter activity compared with the control vector (pGL3-basic), while the ERV insertion significantly enhanced its activity (*p* < 0.01). The luciferase activity in cells transfected with the vector of *TLR6*^ERV+^-Luc (En), which contains the ERV insertion, were almost two times higher (*p* < 0.01) than that in cells transfected with the vector of *TLR6*^ERV—^Luc (En) without the ERV insertion allele in both porcine PK15 and human Hela cells (Fig. 2D), which suggested that the 192 bp ERV may act as an enhancer in the regulation of *TLR6* promoter activity. To further confirm this conclusion, we evaluated the enhancer activity of the ERV insertion by cloning this into the luciferase reporter vector containing a mini-promoter, but without the SV40 enhancer, which is generally used for enhancer activity evaluation. Two types of mini-promoters (β-globin and Oct4) were evaluated, and the outline of the vectors is presented in Fig. 3A and B. Again, the luciferase activity assay revealed that the ERV insertion allele significantly enhances all these mini-promoter activities in both Hela (Fig. 3C) and PK15 cell lines (Fig. 3D) (*p* < 0.05). These data strongly suggest that the 192 bp ERV insertion acts as an enhancer involved in the regulation of *TLRs*.

### ERV insertion alters the expression of pig *TLR6* and *TLR1* and their downstream genes in multiple tissues

**Fig. 4:**
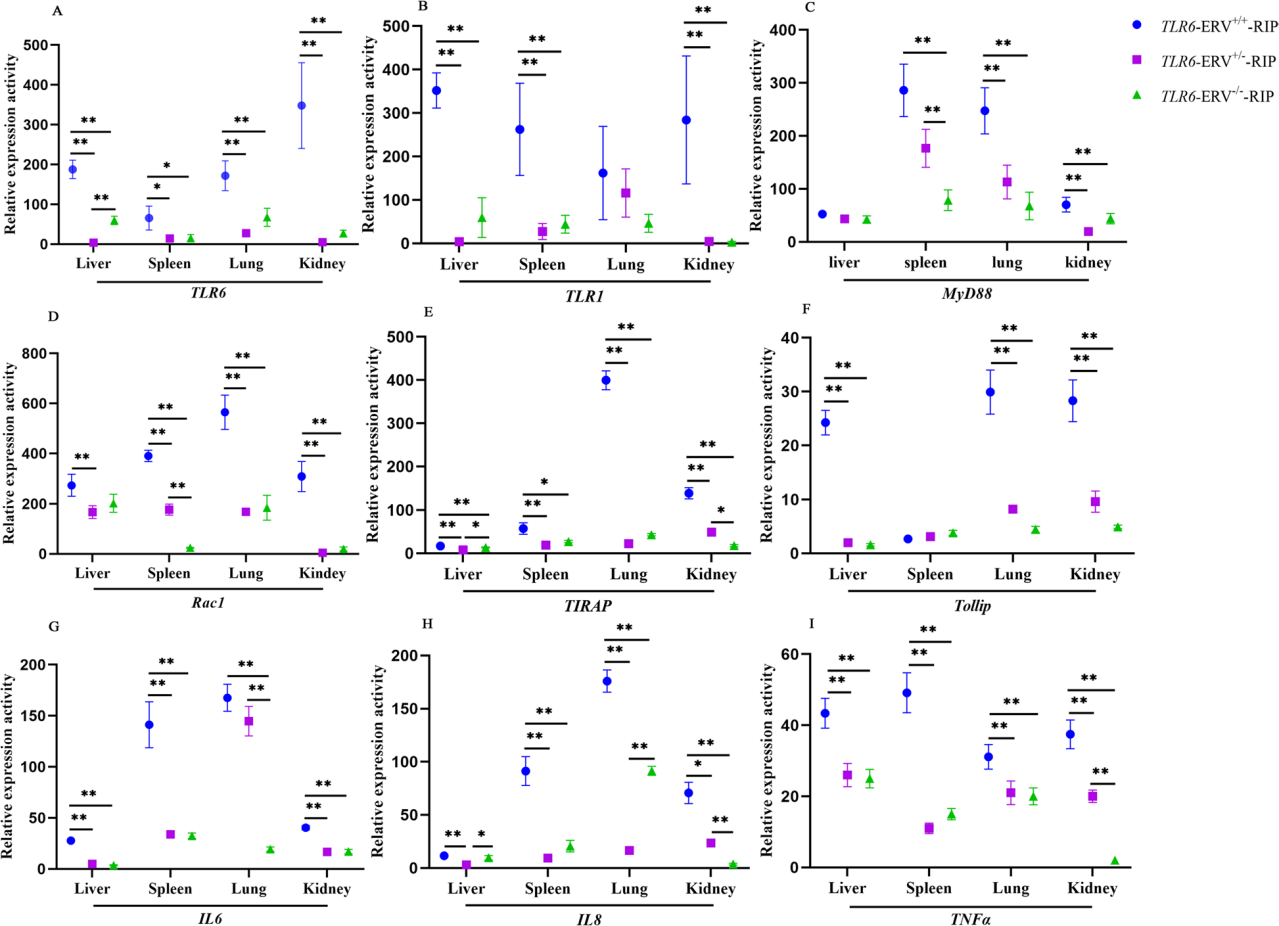
Impact of ERV insertion on expression of *TLR6 *(**A**), *TLR1 *(**B**) *and* downstream genes *MyD88* (**C**), *Rac1* (**D**), *TIRAP* (**E**), *T0llip* (**F**), *IL6* (**G**), *IL8* (**H**), *TNFα* (**I**) of TLR signaling pathway in different tissues of 30-day old piglets. Five piglets for each genotype (ERV^+/+^, ERV^±^, and ERV^−/−^) were selected for qPCR. All measurements were performed in 3 replicates for each individual. *GAPDH* was used to normalize the target genes expression. Values shown are mean ± SD. * showed *p* < 0.05; ** showed *p* < 0.01

To further characterize the biological roles of the 192 bp ERV insertion in the TLR signaling pathway, we investigated the mRNA expression of *TLR6* and *TLR1*, which are neighboring genes located on chromosome 8, and genes downstream in the same pathway (*MyD88*, *Rac1*, *Tollip*, *TIRAP*, *IL6*, *IL8*, and *TNFα*) depending on genotype of the RIP in multiple tissues (liver, spleen, lung and kidney) of 30 day-old piglets using qPCR. The qPCR results revealed that, generally, the ERV insertion was associated with higher expression of *TLR6* and *TLR1* in these tissues. In detail, in the spleen, kidney and liver tissues, the expression of *TLR6* and *TLR1* in the pigs with ERV^+/+^ genotype were significantly higher (*p* < 0.05) than that in those animals with ERV^±^ and ERV^−/−^ genotypes. In lung, there were significant expression differences (*p* < 0.01) of *TLR6* between the ERV^+/+^ animals and the ERV^−/−^ animals (Fig. 4A and 4B). The qPCR analysis of *MyD88*, *Rac1*, *Tollip* and *TIRAP* expression revealed that ERV insertion was associated with enhanced expressions for most of them. In detail, in the spleen, lung and kidney, the expression levels of *MyD88* and *Rac1* were significantly higher (*p* < 0.05) in the animals with ERV^+/+^ than in the animals with ERV^−/−^ genotype (Fig. 4C and 4D). Expression of *TIRAP* and *TOLLip* in liver, lung and kidney of ERV^+/+^ homozygous animals were significantly (*p* < 0.05) higher than those of heterozygote (ERV^±^) and homozygote (ERV^−/−^) animals (Fig. 4E and 4F). In spleen, significant (*p* < 0.05) difference was observed for the expression of *TIRAP* gene between homozygotes ERV^+/+^ and ERV^−/−^ or heterozygote ERV^±^ animals (Fig. 4E). Inflammatory factors *IL6*, *IL8*, *TNFα* are important genes at the end of TLR signaling pathway. The expression of *TNFα*, *IL6*, *IL8* increased significantly (*p* < 0.05) in the spleen, lung and kidney of homozygous of ERV^+/+^ compared with that in ERV^−/−^ genotype piglets (Fig. 4G, 4H, 4I). These results indicated that the 192 bp ERV insertion allele near the core promoter of *TLR6* is associated not only with increased expression of *TLR6* and *TLR1*, but also with the expression levels of the downstream genes of the TLR signaling pathway.

### Impact of ERV insertion on the serum immune cytokine

**Fig. 5:**
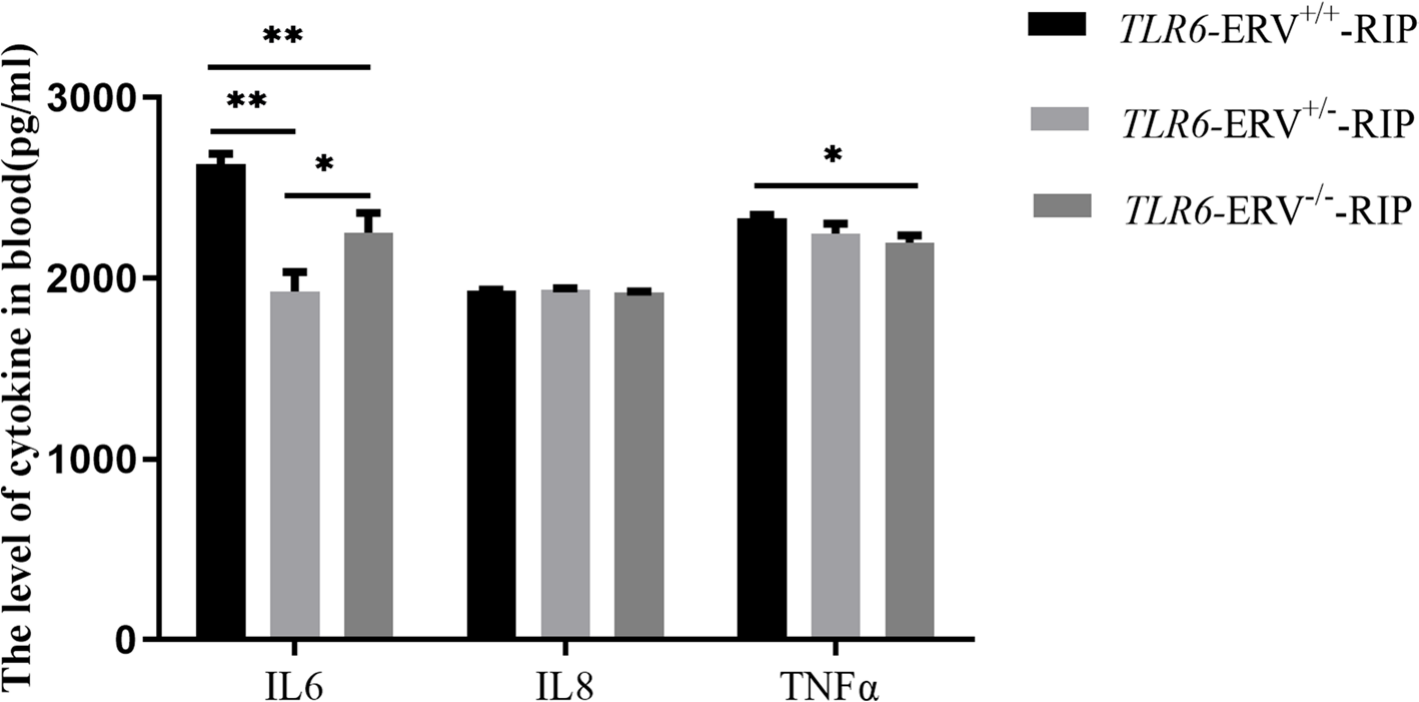
The level of cytokine in blood of different *TLR6-*ERV*-*RIP genotypes. Five piglets for each genotype (ERV^+/+^, ERV^±^, and ERV^−/−^) were selected. All measurements were performed in 4 or 5 replicates for each animal. Values shown are mean ± SD.* showed *p* < 0.05; ** showed *p* < 0.01

To investigate the impact of the ERV insertion allele near the core promoter of *TLR6* on the immune response, several serum immune cytokines were measured by ELISA in 30 day-old piglets. The ELISA analysis revealed that, consistent with the higher expression of *IL6* and *TNFα* in the important immune tissues (spleen and kidney) of ERV^+/+^ piglets compared to other genotypes (ERV^±^ and ERV^−/−^), the serum concentrations of IL6 and TNFα in the animals with ERV^+/+^ genotype were also significantly higher than that in the ERV^−/−^ genotype animals (*p* < 0.05) (Fig. 5). But there is no significant difference of serum IL8 among different genotypes.

## Discussion

Retrotransposons are dominant components in most land plant and mammals genomes [20, 28, 43] and regarded as important drivers of species diversity and putative actors in evolution and adaptation [28, 44–46]. In domestic animals, RIPs have been applied for the analysis of genetic diversity and evolution, and variety identification, and display great potentials in animal genetics and molecular breeding (e.g. sheep [32], chicken [34], and miniature pigs [47]). Our previous study revealed that LINE, LTR, and SINE are the major components in the pig genome, and totally account for about 37.13% of the genome sequence [42]. According to the insertion age analysis, differential evolution profiles were observed for different families and subfamilies of retrotransposons. Most retrotransposons in the pig genome are ancient and no longer jumping, and cannot generate polymorphic insertions in current populations, whereas some of them were thought to be younger retroelements, such as SINEA, L1D, ERV6 subfamilies [42]. These retrotransposons still play roles in shaping genome and gene evolution and contribute to the genomic variations and their insertions tend to generate polymorphisms, which can be used as genetic markers. In addition, it has been suggested that transposable elements affect the genome in both destructive and constructive ways [43]. In this study, we predicted 32 RIPs and finally identified five RIPs in *TLRs* genic and flanking regions by sequence alignment combined with PCR validation and Sanger sequencing. The success rate of experimental validation of the in silico predicted RIPs by PCR was low (15.62%, 5/32). By careful manual inspection of the input sequences we found that the main reason is a deficient assembly of the non-refence genomes, because most predicted RIPs that failed to be confirmed by PCR were located in gaps. Five identified RIPs reside in different genic positions including introns, 5’ and 3’ flanking regions of *TLRs*. Two ERV insertions—one of 192 bp and the other of 413 bp in length—were identified in the first intron of *TLR6* and *TLR7,* respectively. Based on the bioinformatic analysis, the 192 bp ERV insertion in the intron 1 of *TLR6* was predicted to be near the putative core promoter region indicating that it may be involved in gene regulation. In fact, since the ERV LTR contains the U3-R-U5 sequences, which are considered as transcriptional regulators because of the U3 region [48], it may act as an enhancer or promoter [49–51]. Here, the luciferase reporter assay provides experimental evidence supporting potential enhancer function of the 192 bp ERV insertion. Our results show that the ERV element is not only able to increase the *TLR6* promoter activity, but can also enhance the activity of diverse mini-promoters.

Population genetic analysis of the confirmed RIPs revealed that most loci are in Hardy–Weinberg equilibrium, while some polymorphic insertion loci (e.g. *TLR6*-ERV-RIP in Sujiang and Fengjing) show departure, indicating that these may be under selection possibly due to their impact on immune response or other biological function. The deletion allele of *TLR6*-ERV-RIP was found exclusively in Chinese native pig breeds (Bama and Fengjing) or cross breeds containing genetics of Chinese pigs such as Sujiang (Duroc × Jiangquhai × Fengjing), while all analyzed western pig breeds including Landrace, Yorkshire, Duroc, and Sicilian Black from Italy are monomorphic and only contain the ERV insertion allele (ERV^+^). This finding suggest that the deletion allele (ERV^−^) likely originates from Asian pig breeds. Based on the impact of this RIP on immune response as shown by expression analysis of the genes of TLR signaling pathway and serum cytokine measurement (see below), it can be used to improve disease resistance (such as Sujiang) or can be introduced into Chinese native breeds using marker assisted selection. However, the association with immune response still needs further confirmation in a larger cohort.

Piglets used in this study for the analysis of the immune response were 30 days old which represents an important stage for the development of the adaptive immunity in pigs [52]. We used samples from different tissues of these piglets, carrying different ERV insertion genotypes, to evaluate the expression of *TLR6* and *TLR1*, which is a neighboring gene of *TLR6* located only 4.2 kb away. The qPCR analysis demonstrated that the ERV insertion was significantly associated with enhanced mRNA expressions of *TLR6* and *TLR1* in multiple tissues of 30 day-old piglets (*p* < 0.05). *TLRs* play important roles in the innate immune response by recognizing pathogens. They interact with adapter molecules, such as *MyD88, TIRAP, Rac1* and *Tollip*, which are downstream genes of the TLR signaling pathway, to drive the immune responses via the activation of transcription factor NFκB and the production of downstream inflammatory cytokines [53–55]*.* The cytokines, such as IL6 and IL8, released by inflammatory cells are essential factors in resisting pathogen infection. Using qPCR analyses, we further confirmed that the ERV insertion is associated with upregulation of several downstream genes of the TLR signaling pathway in multiple tissues of 30 day-old piglets, suggesting that the ERV insertion may not only increase the expressions of *TLR6* and *TLR1*, but also enhance the expression of their downstream genes.

Higher *TLR1* expression suggested better prognosis in patients with pancreatic ductal adenocarcinoma (*PDAC*) [56]. The mRNA expression of major *TLR* genes including *TLR1* and *TLR6* of Tibetan pigs was higher in most immune tissues compared to Yorkshire pigs, which may result in stronger innate immunity of Tibetan pigs [57]. Higher expression of *TLRs* was also associated with stronger disease resistance [55]. Yorkshire × Landrace (YL) pigs exhibited more serious clinical symptoms when artificially infected with porcine circovirus type 2 (PCV2) virus compared with Laiwu, which is a Chinese native pig breed, indicating YL and Laiwu pigs display different susceptibility to PCV2 infection and Laiwu pigs seem to be more resistant to PCV2 virus. The serum levels of IL6, IL8, IL12 and Transforming growth factor beta 1 (TGFβ1) showed a more pronounced increase at the early infection stages with the PCV2 virus in the Laiwu pigs compared to YL pigs [58]. Here, consistent with the increased expression of genes in the TLR signaling pathway due to ERV insertion, we also found upregulated expression of the important inflammatory factors including *IL6*, *IL8*, and *TNFα*. We validated this finding by measuring serum cytokine levels and found significantly higher concentration of IL6 and TNFα in ERV^+/+^ piglets. Taken together, these data provide evidence that the 192 bp ERV insertion may upregulate the expression of *TLR6, TLR1*, and their downstream genes by acting as an enhancer involved in the regulation of TLR signaling pathway, which may not only alter the gene activities in the TLR signaling pathway and inflammatory factors, but also cause individual variation during the immune response. However, further confirmation of the influence of this allele on genetic and phenotypic variation is highly recommended. In detail, future experiments in additional samples and on a more functional side should be considered to draw a more solid conclusion of the causality. Moreover, association with the phenotypes should be evaluated before determining that this marker should be included in selection schemes for disease resistance.

## Conclusions

By using bioinformatic analysis and PCR-based verification, five RIPs, located in the 3' flanking sequence of *TLR3* gene, 5' flanking region of *TLR5* gene, intron 1 of *TLR6* gene, intron 1 of *TLR7* gene, and 3' flanking region of *TLR8* gene, were identified and differential distribution in diverse pig breeds was observed. The 192 bp ERV insertion in the intron 1 of *TLR6* significantly increases the activity of the *TLR6* promoter and two mini-promoters acting as an enhancer (*p* < 0.05). Furthermore, the ERV insertion was also significantly associated with enhanced expression of *TLR6* and *TLR1*, downstream genes (*MyD88, Rac1*, *TIRAP*, and *Tollip*) of TLR signaling pathway and inflammatory factors (*IL6*, *IL8*, and *TNFα*) in diverse tissues of 30 day-old piglets, as well as higher serum concentrations of IL6 and TNFα (*p* < 0.05). Thus, the 192 bp ERV insertion allele may be beneficial for the immune response and useful for molecular breeding of disease resistant animals.

## Material and methods

### RIP screen

Ten *TLR* genes and their flanking sequences (5 kb 5’ upstream and 3 kb 3’ dowstream) were obtained from fifteen assembled non-reference genomes (Landrace, Yorkshire, Pietrain, Berkshire, Hampshire, Cross-breed of Yorkshire/Landrace/Duroc, Wuzhishan, Tibetan, Rongchang, Meishan, Bamei, Bama, and Jinhua, Goettingen, and Ellegaard Gottingen minipigs) and one reference genome (Duroc) deposited in the NCBI database (https://www.ncbi.nlm.nih.gov/) to screen for structural variations by sequence alignment using the ClustalX program. Large structural variations (more than 50 bp long) present in just one population among sixteen population were retained for further analysis. Retrotransposon (SINE, LINE, and ERV) insertions were annotated by RepeatMasker (http://www.repeatmasker.org/) with a customer constructed library [42]. Promoters were predicted in BDGP (https://fruitfly.org/seq_tools/promoter.html), ENCODE (https://www.encodeproject.org/), and Promoter 2.0 Prediction Server (http://www.cbs.dtu.dk/services/Promoter/). Putative transcription factor binding sites were determined in silico using the online tool PROMO (http://alggen.lsi.upc.es/cgi-bin/promo_v3/promo/promoinit.cgi?dirDB=TF_8.3). The predicted large structural variations (more than 50 bp) overlapping with retrotransposon (SINE, LINE, and ERV) insertions were designated as RIPs. These RIPs were further experimentally validated in seven Chinese native pig breeds (Diannan small-ear Pigs, Erhualian, Wuzhishan, Bama, Tibetan, Meishan, Fengjing Pigs), three commercial pig breeds (Duroc, Landrace, Yorkshire), one cross breed (Sujiang) and wild boars (from Anhui province, Fujian province and Heilongjiang province) by PCR amplification and fragment analysis (Vazyme, Nanjing, China). For each breed, two pooled DNA samples were used, and each pool (50 ng/ul) contained three individual samples. Total DNA was isolated from ear or blood samples of each animal using the TIANamp Genomic DNA Kit (Tiangen, Beijing, China). The quality and concentration of the DNA were measured using a spectrophotometer (NanoPhotometer N60 Touch, Implen Gmbh, Germany) and by running the samples on 0.8% (w/v) electrophoretic gel. Each DNA sample was diluted to 50 ng/ul in concentration for pool mixture. Details on samples and primers used for RIP evaluation are listed in Table S2 and Table S3. All obtained RIPs were further confirmed by TA cloning (Tiangen, Beijing, China) following the manufacturer's instructions and sequencing at TsingKe Bological Technology Co. Ltd (Nanjing, China).

### RIP Genotyping

In total twelve breeds (Duroc, Landrace, Yorkshire, Erhualian, Meishan, Fengjing, Bama, Tibetan, Wuzhishan, Sujiang, and Sicilian black) were used to examine the RIP distribution; the number of animals used for each breed and breed origins is listed in Table S2. Among these breeds, Duroc, Landrace, and Yorkshire are three lean type breeds, Sicilian Black, Erhualian, Meishan, and Fengjing are four fat type pigs and Bama, Wuzhishan, and Tibetan are three miniature pigs. Erhualian, Meishan, Fengjing, Bama, Tibetan, Wuzhishan, are Chinese native breeds, while Sujiang is a synthetic line with 62.5% Duroc, 18.75% Jiangquhai, and 18.75% Fengjing proportion. Sicilian Black is an Italian native breed. The genotype and the allele frequencies were calculated, and Hardy–Weinberg equilibrium were tested using POPGENE [59]. Polymorphic information content (PIC) was calculated by the formula:

$$PIC=1-\sum_{i=1}^{m}{P}_{i}^{2}-\sum_{i=1}^{m-1}\sum_{j=i+1}^{m}2{P}_{i}^{2}{P}_{j}^{2}$$

### Dual luciferase reporter assay

One predicted promoter region (NC_010450.4, 31,167,521–30,168,515) of *TLR6* with (1187 bp) and without the ERV insertion allele (995 bp) was cloned from the Sujiang genomic DNA (primers were listed in Table S3), and verified by sequencing. Then the clones were inserted into the pGL3-basic vectors (Promega, Madison, American) to construct *TLR6*^ERV+^-Luc (En) vector and *TLR6*^ERV−^Luc (En) Vector. In addition, β-globin and Oct4 minipromoters were cloned from pEDV-β-globin-GFP and pTol2-Oct4-mCherry vector, respectively [60] and inserted into the pGL3-basic vectors with or without the 192 bp ERV for enhancer activity evaluation. A total of 2 × 10^4^ PK15 and Hela cells were plated in a 24-well plates and transfected with the plasmids by using Lipofectamine 3000 reagent (Invitrogen, Carlsbad, American). After 48 h, cells were collected for luciferase activity evaluation by using the dual luciferase reporter system (Promega, Madison, American) according to the manufacturer’s protocol.

### Expression analysis

The Sujiang piglets were genotyped and five 30 day-old piglets for each genotype (ERV^+/+^, ERV^±^, and ERV^−/−^) were selected and slaughtered to collect tissue samples including liver, lung, kidney, and spleen. The mRNA samples were extracted and cDNAs were prepared according to the manufacturer’s protocol by using TAKARA Kit (Takara, Tokyo, Japan). Then, the mRNA expressions of *TLR6, TLR1, MyD88, RAC1, Tollip, TIRAP, TNFα, IL6,* and *IL8* mRNA were evaluated by quantitative real-time PCR (qPCR) using the 7900HT Fast Real-Time PCR System (Applied Biosystems, New York, American) in a total volume of 20 μl containing SYBR mix (10 μl), primers (4 ng), and cDNA sample (50 ng) according to the manufacturer’s instructions (Takara,Tokyo, Japan). All measurements were performed in 3 replicates. Glyceraldehyde-3-phosphate dehydrogenase (*GAPDH*) was used as an endogenous control to normalize the target gene expression in four different tissues. Gene expression was measured using the 2^− ΔΔCt^ method. PCR products were run on 1.5% ethidium bromide-stained agarose gels and confirmed using melting curve analyses to assess PCR product quality.

### Measurement of serum TNFα, IL6, and IL8 by enzyme linked immunosorbent assay (ELISA)

TNFα, IL6, IL8 concentration in serum of 30 day-old Sujiang piglets for each genotype (ERV^+/+^, ERV^±^, and ERV^−/−^) were measured using the pig TNFα, IL6, and IL8 ELISA Kit (Solarbio Science, Beijing, China) by following the manufacturer’s protocol. All measurements were performed in 4 or 5 replicates, and mean values were used for statistical analysis.

### Statistical analysis

Experimental results were processed by statistical SPSS17.0 software package (SPSS, Chicago, USA) using one-way analysis of variance with Tukey's post hoc test, and the data were expressed as mean ± SD.

### Animal welfare

All treatments and protocols involving animals in this study were strictly done in accordance with the guidelines of the Animal Experiment Ethics Committee of Yangzhou University (approval number: YZUDWSY2018-12).

## Supplementary Information

**Additional file 1: Table S1**. Predicted Structural variations (SVs) of pig TLRs. **Table S2** Number and origin of pig breeds for TLRs RIPs detection. **Table S3** The primers for PCR, vectors construction and q-PCR.

## Data Availability

All data needed to evaluate the conclusions in this paper are present either in the main text or the Supporting information.

## Abbreviations

TLRs: Toll-like receptors
RIPs: Retrotransposon insertion polymorphisms
QTL: Quantitative trait locus
ERV: Endogenous retrovirus
PCR: Polymerase chain reaction
MyD88: Myeloid differentiation factor 88
Rac1: Rac family small GTPase 1
TIRAP: TIR domain containing adaptor protein
Tollip: Toll interacting protein
IL6: Interleukin 6
IL8: Interleukin 8
TNFα: Tumor necrosis factor alpha
IFN-α: Interferon alpha 1
LTR: Long Terminal Repeat elements
SINE: Short Interspersed Nuclear Elements
ELISA: Enzyme linked immunosorbent assay
IRAP: Inter-retrotransposon amplified polymorphism
REMAP: Retrotransposon-microsatellite amplified polymorphism
NFκB: Nuclear factor kappa B subunit
PDAC: Pancreatic ductal adenocarcinoma
PCV2: Porcine circovirus type 2
TGFβ1: Transforming growth factor beta 1
