# Supplementary material for: A 192 bp ERV fragment insertion in the first intron of porcine TLR6 may act as an enhancer associated with the increased expressions of TLR6 and TLR1
Source: Mob DNA. 2021 Aug 18;12:20. doi: 10.1186/s13100-021-00248-w (PMC8375133; doi:10.1186/s13100-021-00248-w)
Supplement: Supplementary file 1 — Additional file 1: Table S1. Predicted Structural variations (SVs) of pig TLRs. Table S2 Number and origin of pig breeds for TLRs RIPs detection. Table S3 The primers for PCR, vectors construction and q-PCR. [file 13100_2021_248_MOESM1_ESM.docx]

**TableS1 Predicted Structural variations (SVs) of pig *TLRs***

|  | ***TLR1*** | ***TLR2*** | ***TLR3*** | ***TLR4*** | ***TLR5*** | ***TLR6*** | ***TLR7*** | ***TLR8*** | ***TLR9*** | ***TLR10*** | **Total** |
| --- | --- | --- | --- | --- | --- | --- | --- | --- | --- | --- | --- |
| **SVs** | 7 | 7 | 7 | 1 | 8 | 7 | 9 | 2 | 4 | 1 | 53 |
| **SINE** | 1 | 4 | 3 | 1 | 3 | 1 | 0 | 1 | 1 | 0 | 15 |
| **LINE** | 0 | 0 | 0 | 0 | 2 | 0 | 1 | 0 | 2 | 1 | 6 |
| **ERV** | 1 | 0 | 1 | 0 | 0 | 4 | 4 | 0 | 1 | 0 | 11 |
| **Retrotransposons**  **/SVs** (**%)** | 28.58 | 57.14 | 57.14 | 100 | 62.50 | 71.43 | 55.55 | 50 | 100 | 100 | 60.38 |

**TableS2 Number and origin of pig breeds for TLRs RIPs detection**

| Breed | Number | Province/country of origin |
| --- | --- | --- |
| Duroc | 24 | Anhui/China |
| Landrace | 24 |  |
| Yorkshire | 24 |  |
| Landrace | 32 | Dummerstorf/Germany |
| Yorkshire | 31 |  |
| Sicilian black | 30 | Sicily/Italy |
| Sujiang | 163 | Jiangsu/China |
| Diannan small-ear | 6 | Yunnan/China |
| Erhualian | 36 | Jiangsu/China |
| Wuzhishan | 24 | Hainan/China |
| Bama | 43 | Guangxi/China |
| Tibetan | 35 | Sichuan/China |
| Meishan | 24 | Jiangsu/China |
| Fengjing | 24 | Jiangsu/China |
| Wild boars | 12 | Fujian/ Heilongjiang/Anhui /China |

**Table S3 The primers for PCR, vectors construction and q-PCR**

| Name | Forward primer | Reverse primer | Purpose |
| --- | --- | --- | --- |
| *TLR3*-SINE-RIP | AGGCTCTTCCAAATCAAGAAC | GCCCACCTTTGACAAGCTC | RIPs identification |
| *TLR5*-LINE-RIP | CAAAGCGTCTGTGGTCTCAA | CCCACAGCATATGGAGGTTC |  |
| *TLR6-*ERV-RIP | CCAGCTACCAGCTATGTGACTTT | GGTGATATCTCGCTATGACTTTGA |  |
| *TLR7-*ERV-RIP | CTGGGGCCTGAGCCTTTT | GCTCAGATGTGATGGTGCTG |  |
| *TLR8-*SINE-RIP | ACAGGGCCAGGATTCCA | GACTGCTTCATGCATGGTT |  |
| *TLR6*-ERV | CCCAAGCTTGGGAAGGTTTCCTTCGACCCCTA | CGGGGTACCCCGAGCTGCTGAGCTACACCACA | vectors construction |
| ERV192 | CGACGCGTAAAAAAGCAGTATGGTTTCTTGAGGC | GCCCTGACTTTTATGCCCGATATCGAATTCTGCCGAGACCAGCTCAGC(β-globin) |  |
|  |  | AAAGCCTGTTGGCACTGCGATATCGAATTCTGCCGAGACCAGCTCAGC(Oct4) |  |
| β-globin | GCTGAGCTGGTCTCGGCAGAATTCGATATCGGGCATAAAAGTCAGGGC | CGGCTAGCTGTCAGAAGCAAATGTAAGCAATAGA |  |
| Oct4-F | GCTGAGCTGGTCTCGGCAGAATTCGATATCGCAGTGCCAACAGGCTTT | CGGCTAGCGGGGAAGGTGGGCACCCCGA |  |
| *GAPDH* | ATCTTCCAGGAGCGAGATCCC | ATGGTTCACGCCCATCACAA | q-PCR |
| *TLR1* | ATGGTGACAATCAGCAGAGC | TGGTTCCTTTAGCAGCCTTT |  |
| *TLR6* | TTCACCTGGTCTTTCATCCA | GCCCTTGAGTGAGTTCCAAT |  |
| *MyD88* | CAGACCAACTATCGGCTGAA | CAGGGATCAGTCGTTTCTGA |  |
| *RAC1* | CTTATGGGATACGGCTGGAC | GGTGATGGGAGTCAGCTTCT |  |
| *Tollip* | GTCCTGATGCCCACAGTGTA | ATCTGGAGCAGGGAGTTGAT |  |
| *TIRAP* | CCAAGAGCTGGTCTCCTACC | GGAGAGACCTGACATCAACG |  |
| *TNFα* | ACCTCCTCTCTGCCATCAAG | ATAGTCGGGCAGGTTGATCT |  |
| *IL-6* | GATGGCTACTGCCTTCCCTA | GATTGGAAGCATCCGTCTTT |  |
| *IL-8* | TGATTGTGGCAAAGGAGAAG | TTCAAGTCCCCTTTGAGGAG |  |
